# Generative AI-assisted self-visualization and self-concept clarity: evidence consistent with a cognitive-mirroring mechanism in human–AI interaction

**DOI:** 10.3389/fpsyg.2026.1918225

**Published:** 2026-10-28

**Authors:** Yang Guan

**Affiliations:** College of Fine Arts, Shanghai Normal University, Shanghai, China

**Keywords:** cognitive mirroring, generative artificial intelligence, image self-identification, self-concept clarity, self-visualization

## Abstract

Generative artificial intelligence (AI) now lets people turn verbal self-descriptions into visual self-representations, creating a new setting for studying self-concept clarity (SCC) in human–AI interaction. We tested whether generative AI-assisted self-visualization (GAISV) was associated with higher short-term SCC through cognitive mirroring rather than through general immersive flow, and whether this depends on how far participants identify with the generated image. We introduce the AI Mirror Self-Integration Model and evaluated it in two stages. A pilot study (124 valid participants) examined the Cognitive Mirroring in Human-AI Self-Visualization Scale (CM-HAIS) and supported its use as a task-state measure of reflective self-processing. A randomized main experiment then recruited 328 valid participants. Primary analyses compared three conditions—GAISV, active non-self image generation, and passive image viewing (*N* = 246)—with SCC measured immediately after the task and again 48–72 h later (T2 *N* = 216). A self-description-only condition was retained as an exploratory comparison and was not part of the primary tests. GAISV produced higher post-task and follow-up SCC than both control conditions. In parallel mediation models, the GAISV effect was primarily associated with cognitive mirroring rather than with flow, and the cognitive-mirroring indirect path was stronger than the flow path immediately after the task. Image self-identification was associated with the strength of the cognitive-mirroring path at the post-task assessment, as indicated by the index of moderated mediation; the follow-up moderation evidence was weaker. AI-generated self-images were therefore not clarifying by default. GAISV was associated with higher short-term SCC primarily when the image was experienced as personally identifying and engaged reflective cognitive mirroring rather than mere immersion. Condition differences were statistically reliable but small (Cohen’s d ≈ 0.21–0.27), and the mediation results are mechanism-consistent associations rather than evidence of causal ordering.

## 1 Introduction

Self-concept clarity (SCC) is the degree to which a person’s self-beliefs are clearly defined, internally consistent, and stable over time. People high in SCC can readily say who they are and sense that the different parts of the self fit together; low SCC instead goes with uncertainty, fluctuation, and weaker identity integration. Campbell et al. (1996) defined the construct as a structural feature of the self-concept, distinct from transient affect or self-esteem. SCC has since been linked to self-expansion, temporal self-comparison, subjective well-being, and social decision making, marking it as a central resource for self-regulation and adjustment (Emery et al., 2022; Zaw and Baldwin, 2023; Garcia-Arch et al., 2025). Recent studies further show SCC’s relevance for well-being, AI anxiety, and eating-related vulnerability (Antichi et al., 2026; Dong et al., 2026; Li et al., 2026). Because it is relatively stable, brief interactive tasks should not be expected to move it by much. The sharper question is whether a new self-representational context can clarify the self under specific mechanisms and boundary conditions.

Generative AI provides such a context. Traditional social media lets people choose an avatar, post a photograph, or read others’ comments; generative AI goes further, turning a verbal self-description into a visual self-representation. People can thus meet their own self-narratives in an externalized, visual, and quasi-interactive form. Recent work argues that AI is reshaping how people understand identity, introspection, and agency, and that algorithmic platforms increasingly take part in self-making and identity narration (Bhandari and Bimo, 2022; Obreja, 2024; Joseph, 2025). Human-AI interaction reviews likewise emphasize user-centered design, human-AI collaboration, and ethical responsibility when people work with AI agents (Canas, 2022; Jiang et al., 2024; Vaccaro et al., 2024). AI-generated images are also unusual stimuli because their perceived realism and malleability can make them feel like more than ordinary pictures (Miller et al., 2023). In generative AI-assisted self-visualization (GAISV), participants do not simply look at a random image. They first put self-feelings, values, traits, or aspirations into text, and the system renders that text as an image. The result is neither fully self-produced nor unrelated to the self; it is an algorithmically mediated visualization of the participant’s own words. This makes GAISV a useful setting for asking how human-AI interaction shapes self-understanding.

AI-generated self-images should not be assumed to aid self-integration. Work on digital media shows that algorithmic environments can broaden self-experience but can also bring digital stress, social comparison, fragmented self-presentation, and unstable identity (Rafiq and Linden, 2024; Soh et al., 2024; Dodan and Negru-Subtirica, 2025). Generative systems also raise persuasion, disclosure, and privacy concerns because users may treat fluent outputs as personally meaningful feedback (Matz et al., 2024; Register et al., 2025; Reif et al., 2025). AI dialogue can support belief revision in some settings, so the same persuasive capacity should be handled cautiously in self-related contexts (Costello et al., 2024). Highly visual, interactive tasks can also elicit flow-a state of absorption, focused attention, altered time sense, and smooth task experience (Agarwal and Karahanna, 2000; Engeser and Rheinberg, 2008). Evidence from short-video use suggests that flow and social media self-expansion partly explain links between digital media and SCC (Wang and Wang, 2024). This raises a competing account of GAISV. If AI self-images are simply novel, engaging, and immersive, participants may report a pleasant experience that does not actually clarify the self. The effect of GAISV might reflect reflective self-processing, or it might reflect ordinary digital absorption and visual appeal. Unless these paths are separated, it is hard to say whether GAISV changes SCC through a self-clarifying mechanism or through the by-products of an engaging task.

To distinguish these possibilities, we propose cognitive mirroring as the mediating process that links GAISV to SCC. The self is not built through private introspection alone. People also revise self-understanding through external feedback, social mirroring, and reading their own behavioral cues (Bem, 1972; Nass and Moon, 2000; Epley et al., 2007). Work on the digital social mirror likewise treats mediated feedback and platform representations as reference points for identity (Perez-Torres, 2024). Research on chatbot companionship shows that people can form meaningful but uneven relationships with artificial agents, highlighting why self-related AI feedback should be studied as a psychological interaction rather than a neutral display (Chaturvedi et al., 2023; Laestadius et al., 2022; Skjuve et al., 2021). In GAISV, the AI image externalizes a self-description into an object that can be viewed from a slight psychological distance: participants can recognize it as coming from the self while still seeing it as an AI-mediated rendering. We define cognitive mirroring as the reflective identification, distanced observation, and integrative internalization that can occur when a person views an AI-generated self-image. Through it, people may notice self-relevant cues and organize scattered self-feelings more coherently. We do not treat cognitive mirroring as a settled standalone theory; we treat it as a measurable process within this specific human-AI context.

The gap in the literature is not that AI and the self have gone undiscussed. It is that no randomized GAISV study has yet pitted reflective cognitive mirroring directly against immersive flow. Prior work has examined the adaptive value of SCC, identity development online, the algorithmic self, and the realism of AI images (Bennett et al., 2026; Weinmann et al., 2026). Related safety work also shows why AI-generated visuals should be treated cautiously in self-related settings (Craddock et al., 2026). Recent work on AI virtual companions and AI-generated media also links attachment, visual coherence, and suspected artificial origin to self-related or behavioral outcomes (Bunzli and Eppler, 2026; Lebrecht et al., 2026; Liu et al., 2025). AI-generated videos can alter trust through perceived realism and uncanny responses, further supporting the need to separate visual appeal from reflective processing (Liu et al., 2026). Together these strands imply that AI images can influence self-understanding but are unlikely to do so in a uniformly positive or integrative way. An image that does not match a person’s self-description or self-recognition may alienate rather than clarify. Whether GAISV becomes meaningful may therefore hinge on image self-identification-how far participants experience the image as resembling or personally relating to the self. Examining image self-identification as a potential moderator lets us move past the assumption that AI self-images are inherently beneficial, and helps specify when they activate cognitive mirroring instead of staying at the level of visual immersion.

Building on this, we propose the AI Mirror Self-Integration Model (Figure 1), in which GAISV predicts SCC through self-externalization, reflective processing, and image-based self-identification. The study used a two-stage design. A pilot first examined the contextual measurement of cognitive mirroring with the CM-HAIS. A randomized experiment then compared GAISV with an active non-self image-generation control and a passive image-viewing control, with SCC measured immediately after the task and again 48–72 h later. The aim was not to ask whether AI images are generally good for people, but to adjudicate between cognitive mirroring and flow as competing mediators and to test whether image self-identification moderates the cognitive-mirroring path. We tested four hypotheses. H1: GAISV yields higher post-task and short-term follow-up SCC than the active non-self and passive viewing controls. H2: cognitive mirroring mediates the positive link between GAISV and SCC. H3: in a parallel model with both mediators, cognitive mirroring rather than flow is the primary mediating pathway for the association between GAISV and SCC. H4: image self-identification moderates the indirect path from GAISV to SCC through cognitive mirroring, so that the path is stronger when the image feels more personally identifying.

**Figure 1:**
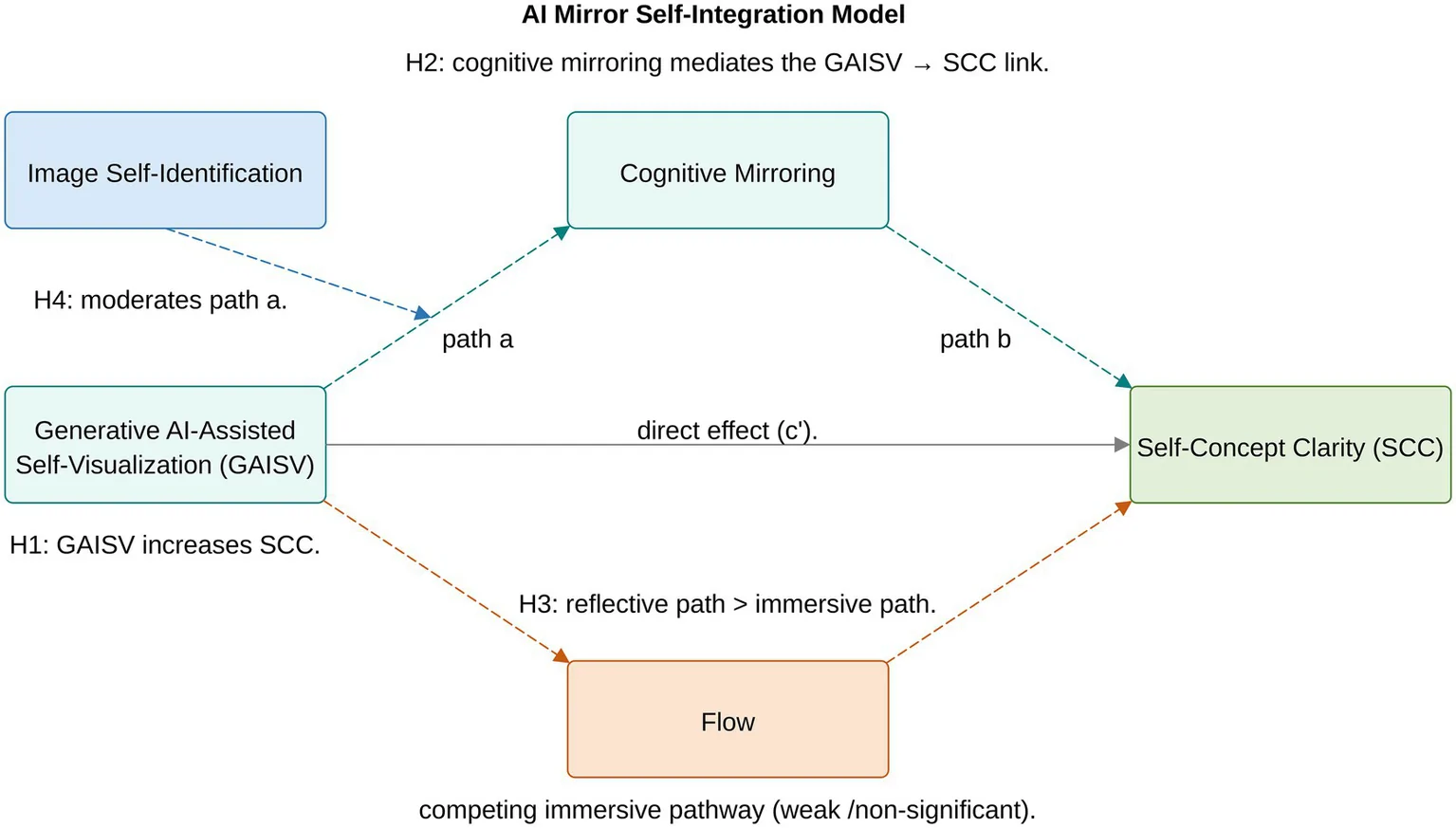
AI Mirror Self-Integration Model. The model proposes that generative AI-assisted self-visualization predicts self-concept clarity through cognitive mirroring. Flow is modeled as a competing immersive pathway rather than a negative mechanism, and image self-identification is specified as a hypothesized moderator of the cognitive-mirroring pathway, measured after the task. Line style indicates the evidential status of each path: The solid arrow denotes the path identified by random assignment (condition → self-concept clarity), whereas dashed arrows denote hypothesized or observationally estimated process paths (cognitive mirroring, image self-identification, and flow), which were measured after the task rather than experimentally manipulated and therefore provide correlational process evidence.

## 2 Materials and methods

### 2.1 Participants and design

The study had two stages: a pilot and a randomized main experiment. The pilot evaluated the contextual measurement of cognitive mirroring in a GAISV setting. Of 132 pilot responses, 124 passed the attention-check and data-quality criteria and were retained. The pilot focused on the CM-HAIS alongside adjacent constructs used for preliminary convergent and discriminant evidence: task-state flow, trait self-reflection, private self-consciousness, and rumination (Trapnell and Campbell, 1999).

The main experiment used an online randomized design. The raw platform export held 376 responses; after the pre-specified data-quality criteria, the final effective sample was 328. The platform dataset contained four conditions: GAISV, active non-self image generation, passive image viewing, and self-description only. The primary tests of H1–H4 used the three conditions central to the mechanism-adjudication model-GAISV (*n* = 80), active non-self image generation (*n* = 84), and passive image viewing (*n* = 82)-for a primary analysis sample of *N* = 246. The self-description-only condition (*n* = 82) was kept as an exploratory comparison to help separate self-description from AI-mediated self-visualization; it was neither merged with passive viewing nor included in the primary tests. At the 48–72 h follow-up, 221 participants in the three primary-analysis groups completed T2, and 216 met the pre-specified T2 analysis criteria after follow-up attention and data-quality checks. The sample size was not determined by a formal *a priori* power analysis. To evaluate whether the achieved sample was adequate, we conducted a sensitivity power analysis for the primary ANCOVA (three groups, five covariates, α = 0.05, power = 0.80), computed from the noncentral F distribution as implemented in G*Power (Faul et al., 2007). The primary analysis sample (N = 246) could detect condition effects of *f* = 0.20 (partial η^2^ = 0.038) or larger at T1, and the T2 analysis sample (N = 216) effects of *f* = 0.21 (partial η^2^ = 0.043) or larger; the observed condition effects (partial η^2^ = 0.050 and 0.057; see Results) exceeded these thresholds, although smaller effects could have gone undetected.

Participants were eligible if they were at least 18 years old, could read Chinese fluently (the study was conducted in Chinese), and gave informed consent. Recent generative-AI experience was an eligibility requirement: participants were included only if they reported having used a generative AI tool at least once in the past 30 days, ensuring they could complete the task. The degree of prior experience (familiarity, recent frequency, prior image-generation or editing experience, and number of uses in the past 30 days) was additionally measured and entered as a covariate rather than used to screen. Responses were linked by de-identified IDs. We excluded cases for missing consent, failed screening, failed attention checks, incomplete main-session data, missing key variables, task non-compliance, extreme completion time, duplicate or invalid records, and failed or unusable image generation where generation was required. Attention checks were embedded at T0, T1, and T2. Respondent-style diagnostics, such as straight-lining and dominant within-scale response patterns, were recorded for sensitivity checks but did not alter the effective sample once the main exclusion rules had been applied. The overall participant flow (raw platform export *N* = 376 to effective sample *N* = 328) and condition-wise follow-up (T2) retention are reported in the Supplementary material; because per-case exclusion reasons were not retained as fields in the de-identified analysis dataset, exclusions are summarized at the criterion level overall rather than cross-tabulated by condition and criterion.

Both the pilot and the main experiment were conducted on the online research platform Credamo, using its participant panel, which supports eligibility screening, quota control, automated random assignment to conditions, timed multi-wave (T0-T1-T2) delivery, unique-ID matching across waves, and raw response export. Recruitment was open to registered adult panel members who met the eligibility criteria (age > = 18, fluent Chinese reading, informed consent, and recent generative-AI use); no student subject pool or offline convenience sampling was used. The pilot (132 responses, 124 retained) and the main experiment (376 responses, 328 retained) were drawn from the same panel and screened with identical criteria but were independent, non-overlapping samples collected in separate waves; pilot participants were excluded from the main experiment at recruitment via panel-level de-duplication by unique member ID, so no individual contributed to both stages, and the pilot served solely to validate the CM-HAIS.

Participants were compensated through the platform’s standard payment system after submitting a valid, quality-passing response, independent of the content or direction of their responses. Pilot participants received a one-time payment of CNY 6 (about US$0.85) for a single session (mean approximately 9 min). In the main experiment, participants received CNY 10 (about US$1.40) for the T0-T1 session (mean approximately 14 min) and a CNY 6 (about US$0.85) bonus for completing the 48–72 h T2 follow-up, for up to CNY 16 (about US$2.20) in total.

### 2.2 Procedure and manipulation

The experiment followed a T0–T1–T2 structure (Figure 2). At T0, participants completed demographics, generative AI experience, baseline SCC, self-esteem, and trait self-reflection. Demographics covered age, gender, education, occupation, and country or region. Generative AI experience items assessed familiarity, recent frequency of use, prior AI image-generation or editing experience, and number of uses in the previous 30 days.

**Figure 2:**
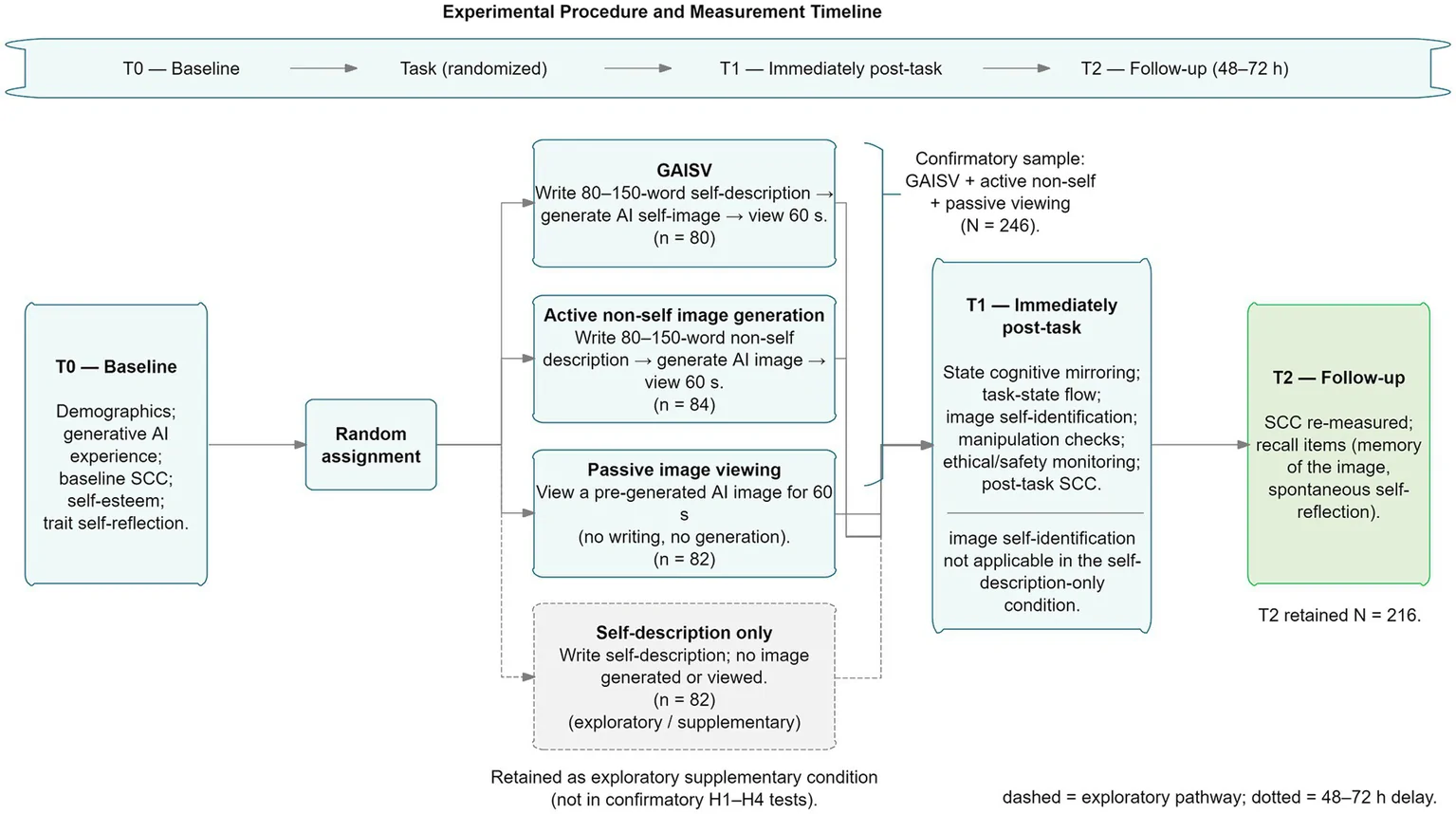
Experimental procedure and measurement timeline. The figure summarizes the T0–T1–T2 design: Baseline measures, randomized task assignment, image generation or viewing, immediate post-task measures, and the 48–72 h follow-up. The primary model used the GAISV, active non-self image generation, and passive image viewing conditions; the self-description-only condition was retained as an exploratory supplementary condition.

After T0, the platform randomly assigned participants to a condition. In the GAISV condition, participants wrote an 80–150 word self-description of characteristics, values, interests, current situation, or future direction that felt representative of them; this text generated an AI self-image, which they viewed for 60 s before the post-task measures. In the active non-self control, participants instead wrote an 80–150 word description of a fictional person or neutral target unrelated to themselves, generated an AI image from it, and viewed it for 60 s. In the passive image-viewing control, participants viewed a pre-generated AI image for 60 s without writing a description or generating an image. In the self-description-only exploratory condition, participants completed the self-description task but did not generate or view an AI self-image.

Immediately after the task, participants completed the T1 measures: state cognitive mirroring, task-state flow, image self-identification, manipulation checks, ethical safety monitoring, and post-task SCC. Manipulation checks covered task self-relevance, task engagement, image quality, perceived realism, and discomfort. Image-specific measures (image self-identification, image quality, and perceived realism) were shown only in the three image conditions. Participants in the self-description-only condition, which involved no image, completed the remaining T1 measures, including cognitive mirroring, flow, the task self-relevance, engagement, and discomfort checks, and post-task SCC. Participants were then invited to a follow-up 48–72 h later. At T2, SCC was measured again with the same items, and participants completed brief recall items on whether they remembered the AI image and whether they had spontaneously thought about it or related self-reflections since the session.

The three image conditions were designed to isolate distinct components of GAISV. The active non-self condition holds constant the writing, generation, and image-viewing components while removing self-relevance; the passive-viewing condition removes both writing and generation; and the self-description-only condition (exploratory) retains self-description while removing image generation and viewing. Comparing GAISV with the passive- and active-control conditions therefore isolates the added value of self-relevant AI visualization over mere viewing and over non-self generation, whereas the exploratory GAISV versus self-description-only comparison indexes the increment attributable to the AI-generated image beyond self-description alone.

AI images were generated with OpenAI’s image-generation feature in ChatGPT (“ChatGPT Images”), accessed through the ChatGPT web interface, between 23 June 2025 and 13 March 2026. Because the web interface did not expose a fixed model version, the model or version identifier visible at generation was recorded where available rather than treated as a fixed experimental constant. The prompt template and generation procedure were standardized across participants. Each participant in an image condition ultimately used a single image. In the GAISV condition, one self-representation image was generated from the participant’s self-description; in the active non-self condition, one image was generated from a non-self, neutral description; in the passive viewing condition, participants viewed a pre-generated, style-matched neutral image; and in the self-description-only condition, participants wrote a self-description but generated no image.

Several safeguards protected participant privacy and content safety. Participants did not upload real face photographs and were not asked for real names, school or company affiliations, addresses, or contact details. The retained dataset includes de-identified participant ID, condition, the model/version identifier and generation date/time only where the interface exposed and logged them (these were not systematically available), de-identified prompt-text fields, image ID, image-generation success status, and image-quality or image-identification variables. Broader safety, deletion-request, and non-compliance decisions were handled as study-procedure records and should be supplied as controlled-access supplementary documentation if requested, rather than overstated as fully public row-level fields. Prompt templates, generation parameters where available, and non-identifying example images are provided or can be supplied in the Supplementary material.

### 2.3 Measures

All study materials—the consent form, instructions, the self-description task, all questionnaires, and the AI image prompts—were administered in Chinese, and participants were required to read Chinese fluently. For the Self-Concept Clarity Scale and the Rosenberg Self-Esteem Scale, the Chinese items were taken from previously published Chinese versions (Niu et al., 2016; Peng et al., 2024). For the brief self-reflection and flow measures, whose published Chinese versions differ in length and context from the short adapted forms used here, the selected items were translated from the original English using a forward-translation and back-translation procedure, with published Chinese versions consulted for reference (Chen et al., 2016; Liu et al., 2012); the Chinese wording was reviewed by bilingual researchers and checked for clarity during the pilot. The CM-HAIS and the image self-identification scale were developed in Chinese for this study. Unless noted, attitudinal items used a 7-point Likert scale from 1 (strongly disagree) to 7 (strongly agree). Scale scores were item means after any reverse scoring, with higher scores indicating more of the construct.

Self-concept clarity was measured at T0, T1, and T2 with a 12-item adaptation of the Self-Concept Clarity Scale (SCCS; Campbell et al., 1996), tapping the clarity, stability, and internal consistency of self-beliefs. Items SCC3, SCC5, SCC8, and SCC10 were reverse scored. Using the same items at all three time points let us compare baseline, immediate post-task, and follow-up SCC while controlling for baseline differences.

Cognitive mirroring was measured at T1 with the 7-item CM-HAIS, developed for this human–AI self-visualization context to capture reflective processing, distanced self-observation, and integrative internalization after viewing an AI image. Sample content included seeing oneself from a new perspective, observing oneself from the outside, connecting scattered self-feelings, and organizing one’s self-understanding more clearly. CM6 was reverse scored, as it indicated that the image prompted no self-related thought. The pilot examined the scale’s preliminary structure and validity. We treat CM-HAIS as a contextual operationalization of a mediating process, not as an established general-purpose trait scale.

Task-state flow was measured at T1 with a 5-item task-adapted flow scale drawn from prior flow and optimal-experience research (Jackson and Marsh, 1996; Engeser and Rheinberg, 2008), assessing engagement, altered time perception, absorption, task fluency, and focused attention. Flow served as a competing mediator because it indexes immersive involvement without tapping self-reflection or self-organization.

Image self-identification was measured at T1 with a 5-item scale assessing how far the AI image felt personally identifying—whether it matched the self-description, reflected the participant’s feelings about the self, could be seen as a self-related representation, and felt personally relevant. ISI5 was reverse scored, as it stated that the image did not look like or represent the participant. Image self-identification was the moderator in H4 and was kept conceptually distinct from image quality, perceived realism, and task-level self-relevance.

Self-esteem was assessed at T0 with a 4-item brief Rosenberg measure (Rosenberg, 1965); RSE3 was reverse scored. Trait self-reflection was assessed at T0 with a 4-item brief measure adapted from self-reflection and insight research (Grant et al., 2002); SR4 was reverse scored. Both were included as covariates because baseline self-evaluation and dispositional reflection may shape SCC and responses to self-visualization. Generative AI experience was assessed through familiarity, recent use, image-generation experience, and use frequency. Age was also a covariate in the primary models.

Additional task and quality variables supported manipulation checks and diagnostics. Self-description quality (SDQ) indexed the personalization and specificity of the description. Manipulation checks assessed task self-relevance (MC1), task engagement (MC2), image quality (MC3), perceived realism (MC4), and discomfort (MC5). Further image diagnostics included AI image self-similarity, image satisfaction, image unfamiliarity, self-discrepancy, generation quality, generated-image personalization, and post-task reflection depth. Attention-check items required pre-specified responses at T0, T1, and T2. Open-ended post-task reflective writing was collected for supplementary or exploratory analysis and was not part of the primary H1–H4 model.

### 2.4 Analytic strategy, ethics, and transparency

Primary analyses followed the four hypotheses and were distinguished from exploratory comparisons. H1 used analysis of covariance (ANCOVA) predicting T1 and T2 SCC from condition, with baseline SCC, self-esteem, trait self-reflection, generative AI experience, and age as covariates. The condition variable comprised GAISV, active non-self, and passive viewing, and pairwise contrasts compared GAISV with each control. The self-description-only group was described and kept for supplementary exploration but excluded from the primary models.

This study was not publicly preregistered. The four hypotheses (H1–H4), the exclusion criteria, the primary outcomes, the covariate set, and the decision to treat the self-description-only condition as exploratory were specified prior to data analysis; we therefore describe these as planned primary analyses rather than as preregistered primary tests, and a written analysis plan can be provided on request.

H2 used bootstrap mediation with GAISV predicting SCC through cognitive mirroring. H3 used a parallel mediation model with cognitive mirroring and flow as simultaneous mediators, estimating each indirect effect, the direct effect, and the contrast between the two indirect effects, which tested whether the reflective path outweighed the immersive path. Indirect effects used bootstrap 95% confidence intervals, following recommendations for multiple-mediator models (Preacher and Hayes, 2008). Because the T1 mediators and T1 SCC came from the same post-task session, we interpret the mediation models as mechanism-consistent statistical tests rather than evidence of temporal causal ordering among post-task states. In all mediation and moderated mediation models, condition was coded as GAISV = 1 versus the two image-control conditions combined = 0; “GAISV” in these models therefore denotes the contrast between GAISV and the active non-self and passive-viewing conditions, which did not differ in SCC at either time point. Every equation included the same five covariates as the ANCOVA (baseline SCC, self-esteem, trait self-reflection, generative AI experience, and age). To show the effect relative to each control condition, we additionally estimated relative indirect effects using indicator coding of the three-category condition variable (Hayes and Preacher, 2014), with passive viewing and, in a second model, active non-self as the reference group.

H4 used moderated mediation, with image self-identification moderating the path from GAISV to cognitive mirroring. The models estimated the GAISV × image self-identification interaction, conditional indirect effects at low, mean, and high image self-identification, and the index of moderated mediation (Hayes, 2015), which served as the primary test of H4. Because the study included a short-term follow-up, the parallel and moderated mediation models were repeated with T2 SCC as the outcome, controlling for T0 SCC and the same covariates. Image self-identification was standardized within each analysis sample before the interaction term was formed, and the outcome equation included both mediators and image self-identification.

Reliability and item-quality diagnostics were computed for the core scales. For the pilot, we examined CM-HAIS structure, internal consistency, and correlations with adjacent constructs. For the main experiment, we computed reliabilities, item loadings, descriptive statistics, and correlations for SCC, CM-HAIS, flow, image self-identification, self-esteem, and trait self-reflection, and inspected reverse-scored items, loading differences, and possible item noise. Respondent-style indicators and subgroup diagnostics served as supplementary robustness checks and did not redefine the primary analysis sample after exclusions.

We additionally report discriminant-validity analyses for the CM-HAIS (confirmatory factor analysis, average variance extracted, and heterotrait–monotrait ratios against SCC and adjacent constructs), a test of differential attrition at follow-up, a preplanned exploratory contrast between the GAISV and self-description-only conditions, and an additional exploratory parallel mediation model with self-description only as the baseline. Indirect effects in these additional analyses used 5,000 percentile bootstrap resamples.

The study was a low-risk online behavioral experiment. Participants gave informed consent before starting and were told that the study involved AI image generation or viewing, self-description, questionnaires, and follow-up contact; that participation was voluntary; that they could withdraw at any time; and that they could request deletion of generated images and data. The AI image task did not require uploading real face photographs, generation was text-based, and analytic data were stored under de-identified IDs (see Ethics Statement).

The project followed a transparency-oriented workflow, retaining the raw platform export, effective-sample dataset, variable dictionary, analysis scripts, model outputs, prompt-template records, and figure data in the project archive. The analysis plan separated the three-condition primary analysis sample from the exploratory self-description-only condition. Because participant-generated prompts and AI images may contain identifying or sensitive self-related material, the Data Availability Statement specifies controlled access for de-identified data, code, statistical outputs, and figure data.

## 3 Results

### 3.1 Pilot validation and descriptive statistics

The pilot validation sample comprised 124 participants who passed the attention-check and data-quality criteria. The 7-item CM-HAIS showed acceptable internal consistency (Cronbach’s α = 0.846). A one-factor extraction gave a first eigenvalue of 3.703, with item loadings from 0.579 to 0.808. Adjacent measures showed acceptable to high reliability: flow, α = 0.842; trait self-reflection, α = 0.761; private self-consciousness, α = 0.862; rumination, α = 0.866. CM-HAIS correlated positively but not redundantly with these constructs—flow (r = 0.142), trait self-reflection (r = 0.235), private self-consciousness (r = 0.372), and rumination (r = 0.200)—supporting its retention as the task-state measure of cognitive mirroring in the main experiment.

The main experiment retained 328 valid participants after exclusions. Primary analyses used the three planned conditions (*N* = 246): GAISV (*n* = 80), active non-self image generation (*n* = 84), and passive image viewing (*n* = 82). The self-description-only condition (*n* = 82) was retained for descriptive and supplementary purposes only. Among the three primary-analysis groups, 216 participants met the T2 criteria, with retained analysis samples of 76 (GAISV), 70 (active non-self), and 70 (passive viewing). Table 1 reports participant characteristics and manipulation-check variables by condition, including the exploratory self-description-only group, which was not part of the primary models.

**Table 1:** Participant characteristics and focal manipulation-check variables by condition.

Condition	n	Age, M (SD)	Female %	AI exp., M (SD)	T0 SCC, M (SD)	T1 SCC, M (SD)	CM, M (SD)	Flow, M (SD)	ISI, M (SD)	Self-rel., M (SD)	Engage., M (SD)	T2 retained, n (%)
GAISV	80	31.70 (9.62)	42.5	4.54 (0.90)	4.65 (0.80)	4.84 (0.85)	4.51 (0.78)	4.43 (0.90)	4.86 (0.83)	5.66 (0.90)	4.90 (0.76)	76 (95.0)
Active non-self image generation	84	32.20 (8.56)	41.7	4.54 (0.93)	4.58 (0.85)	4.60 (0.96)	4.07 (0.71)	4.35 (0.78)	3.09 (0.82)	3.19 (0.91)	4.81 (0.83)	70 (83.3)
Passive image viewing	82	31.43 (9.77)	52.4	4.63 (0.99)	4.64 (0.70)	4.59 (0.87)	4.02 (0.74)	4.00 (0.76)	3.15 (0.81)	2.91 (0.88)	4.56 (0.79)	70 (85.4)
Self-description only	82	30.39 (8.72)	42.7	4.29 (0.79)	4.66 (0.94)	4.73 (1.08)	4.32 (0.77)	4.38 (0.79)	N/A	5.27 (0.85)	4.82 (0.80)	70 (85.4)

GAISV, generative AI-assisted self-visualization; AI exp., generative AI experience; SCC, self-concept clarity; CM, cognitive mirroring; ISI, image self-identification; Self-rel., task self-relevance; Engage., task engagement. The three primary-analysis groups were GAISV, active non-self image generation, and passive image viewing (*N* = 246). The self-description-only group is reported for descriptive and supplementary purposes only and was not included in the primary H1–H4 models. ISI was not applicable in the self-description-only condition because no AI image was generated or viewed. Additional manipulation checks for image quality, perceived realism, discomfort, and completion time are reported in the Supplementary material.

Reliability was adequate across core measures in the primary analysis sample. SCC was highly consistent at T0 (α = 0.915), T1 (α = 0.932), and T2 (α = 0.939). Other reliabilities were: CM-HAIS, α = 0.878; flow, α = 0.833; image self-identification, α = 0.917; self-esteem, α = 0.761; trait self-reflection, α = 0.750. Item loadings fell within acceptable ranges: SCC at T0, 0.573–0.807; SCC at T1, 0.588–0.829; SCC at T2, 0.658–0.848; CM-HAIS, 0.658–0.820; flow, 0.745–0.808; image self-identification, 0.790–0.904. Descriptive statistics were: SCC at T0, M = 4.623, SD = 0.784; SCC at T1, M = 4.674, SD = 0.899; SCC at T2, M = 4.644, SD = 0.947; cognitive mirroring, M = 4.197, SD = 0.773; flow, M = 4.259, SD = 0.829; image self-identification, M = 3.683, SD = 1.155; self-esteem, M = 4.794, SD = 0.757; trait self-reflection, M = 4.593, SD = 0.807; AI experience, M = 4.566, SD = 0.940. Full descriptives, reliabilities, and correlations appear in Table 2.

**Table 2:** Descriptive statistics, reliability coefficients, item-loading ranges, and zero-order correlations for core study variables.

Variable	*n*	M	SD	α	Loading range	1	2	3	4	5	6	7	8
1. SCC T0	246	4.623	0.784	0.915	0.573–0.807	—							
2. SCC T1	246	4.674	0.899	0.932	0.588–0.829	0.854	—						
3. SCC T2	216	4.644	0.947	0.939	0.658–0.848	0.864	0.817	—					
4. Cognitive mirroring	246	4.197	0.773	0.878	0.658–0.820	0.179	0.362	0.276	—				
5. Flow	246	4.259	0.829	0.833	0.745–0.808	0.133	0.195	0.203	0.188	—			
6. Image self-identification	246	3.683	1.155	0.917	0.790–0.904	0.008	0.161	0.093	0.393	0.146	—		
7. Self-esteem	246	4.794	0.757	0.761	0.698–0.793	0.387	0.315	0.410	0.087	0.030	−0.036	—	
8. Trait self-reflection	246	4.593	0.807	0.750	0.623–0.824	0.152	0.177	0.195	0.244	0.090	0.023	0.030	—
9. AI experience	246	4.566	0.940	—	—	0.023	−0.033	0.023	−0.006	−0.123	0.080	0.047	−0.028

SCC, self-concept clarity. Values are based on the three-condition primary analysis sample unless otherwise indicated; T2 SCC was available for the retained follow-up sample (*n* = 216). Scale reliabilities are Cronbach’s α, and loading ranges are from one-factor item diagnostics. Reverse-scored items were SCC3, SCC5, SCC8, and SCC10 at each SCC time point; CM6; ISI5; RSE3; and SR4.

Discriminant validity of cognitive mirroring. Because CM-HAIS items and SCC items both refer to organizing self-relevant material, we tested whether the two were empirically separable. A two-factor confirmatory model distinguishing cognitive mirroring from post-task SCC fit well, χ^2^(151) = 159.6, CFI = 0.997, TLI = 0.996, RMSEA = 0.015, SRMR = 0.035, and fit far better than a one-factor model combining them, χ^2^(152) = 768.1, CFI = 0.758, RMSEA = 0.129 (Δχ^2^(1) = 608.5). The latent correlation was moderate (r = 0.388); the square roots of the average variance extracted (CM = 0.720; SCC = 0.745) exceeded it (Fornell–Larcker criterion), and the heterotrait–monotrait ratio was well below conventional thresholds (HTMT = 0.398). In the pilot sample, cognitive mirroring was similarly distinguishable from trait self-reflection (HTMT = 0.296), private self-consciousness (HTMT = 0.435), and rumination (HTMT = 0.241). Cognitive mirroring is therefore related to but distinct from SCC and adjacent reflective constructs rather than a relabeling of the outcome.

### 3.2 Manipulation checks and main effect

Manipulation checks showed the expected differences in self-relevance and image self-identification while image quality and discomfort stayed comparable across image conditions. For task self-relevance (MC1), GAISV exceeded passive viewing, B = 2.748, SE = 0.141, 95% CI [2.472, 3.024], *p* < 0.001, and active non-self image generation, B = 2.472, SE = 0.140, 95% CI [2.198, 2.746], *p* < 0.001; active non-self was slightly above passive viewing, B = 0.276, SE = 0.139, 95% CI [0.003, 0.549], *p* = 0.049. For task engagement (MC2), GAISV exceeded passive viewing, B = 0.339, SE = 0.124, 95% CI [0.095, 0.583], *p* = 0.007, but did not differ from active non-self, B = 0.090, SE = 0.124, 95% CI [−0.152, 0.333], *p* = 0.465. Image quality (MC3), perceived realism (MC4), and discomfort (MC5) did not differ significantly between GAISV and either control.

The manipulation also separated the process variables. GAISV produced higher cognitive mirroring than passive viewing, B = 0.490, SE = 0.117, 95% CI [0.260, 0.719], *p* < 0.001, and than active non-self, B = 0.439, SE = 0.116, 95% CI [0.211, 0.667], *p* < 0.001; the two controls did not differ, B = 0.051, SE = 0.116, 95% CI [−0.176, 0.277], *p* = 0.663. Flow was higher in GAISV than passive viewing, B = 0.432, SE = 0.127, 95% CI [0.183, 0.682], *p* < 0.001, and higher in active non-self than passive viewing, B = 0.352, SE = 0.126, 95% CI [0.106, 0.599], *p* = 0.006, while GAISV and active non-self did not differ, B = 0.080, SE = 0.127, 95% CI [−0.168, 0.328], *p* = 0.528. Image self-identification was higher in GAISV than passive viewing, B = 1.704, SE = 0.129, 95% CI [1.451, 1.957], *p* < 0.001, and than active non-self, B = 1.769, SE = 0.128, 95% CI [1.518, 2.021], *p* < 0.001; the two controls did not differ, B = −0.066, SE = 0.127, 95% CI [−0.315, 0.184], *p* = 0.608. Flow thus rose with both image-generation tasks, whereas cognitive mirroring and image self-identification rose specifically with self-relevant generation.

H1 was tested with ANCOVA on post-task and follow-up SCC, controlling for baseline SCC, self-esteem, trait self-reflection, AI experience, and age. At T1, the omnibus condition effect was significant, *F*(2, 238) = 6.20, partial η^2^ = 0.050, *p* = 0.002. GAISV produced higher T1 SCC than passive viewing, B = 0.241, 95% CI [0.100, 0.382], *p* < 0.001, and than active non-self, B = 0.191, 95% CI [0.050, 0.332], *p* = 0.008; the two controls did not differ, B = 0.050, 95% CI [−0.090, 0.190], *p* = 0.483. At T2, the omnibus effect was again significant, *F*(2, 208) = 6.28, partial η^2^ = 0.057, *p* = 0.002, with GAISV above passive viewing, B = 0.227, 95% CI [0.079, 0.374], *p* = 0.003, and above active non-self, B = 0.232, 95% CI [0.085, 0.380], *p* = 0.002; the controls did not differ, B = −0.006, 95% CI [−0.156, 0.145], *p* = 0.941. These effects were consistent with H1, although the partial η^2^ values indicate that the condition differences, while reliable, were modest in size. Table 3 summarizes the experimental effects. Standardized, these condition differences were small (Cohen’s d ≈ 0.21–0.27 for the GAISV contrasts).

**Table 3:** Experimental effects, parallel mediation, and moderated mediation results.

Analysis	Estimate	95% CI/effect size	*p*
T1 SCC ANCOVA condition omnibus	*F*(2, 238) = 6.20	partial η^2^ = 0.050	0.002
T1 SCC: GAISV—passive viewing	0.241	[0.100, 0.382]	< 0.001
T1 SCC: active non-self—passive viewing	0.050	[−0.090, 0.190]	0.483
T1 SCC: GAISV—active non-self	0.191	[0.050, 0.332]	0.008
T2 SCC ANCOVA condition omnibus	*F*(2, 208) = 6.28	partial η^2^ = 0.057	0.002
T2 SCC: GAISV—passive viewing	0.227	[0.079, 0.374]	0.003
T2 SCC: active non-self—passive viewing	−0.006	[−0.156, 0.145]	0.941
T2 SCC: GAISV—active non-self	0.232	[0.085, 0.380]	0.002
T1 mediation: GAISV → CM	0.494	[0.302, 0.692]	—
T1 mediation: CM → SCC	0.226	[0.155, 0.299]	—
T1 mediation: indirect via CM	0.112	[0.062, 0.173]	—
T1 mediation: GAISV → flow	0.258	[0.041, 0.499]	—
T1 mediation: flow → SCC	0.038	[−0.031, 0.111]	—
T1 mediation: indirect via flow	0.010	[−0.007, 0.038]	—
T1 mediation: indirect contrast, CM—flow	0.102	[0.044, 0.168]	—
T1 mediation: direct effect	0.094	[−0.016, 0.210]	—
T2 mediation: GAISV → CM	0.526	[0.324, 0.725]	—
T2 mediation: CM → SCC	0.088	[0.004, 0.168]	—
T2 mediation: indirect via CM	0.046	[0.002, 0.096]	—
T2 mediation: GAISV → flow	0.290	[0.054, 0.530]	—
T2 mediation: flow → SCC	0.027	[−0.054, 0.108]	—
T2 mediation: indirect via flow	0.008	[−0.015, 0.040]	—
T2 mediation: indirect contrast, CM—flow	0.038	[−0.017, 0.094]	—
T2 mediation: direct effect	0.175	[0.036, 0.307]	—
T1 moderated mediation: GAISV × ISI → CM	0.338	[0.072, 0.597]	—
T1 moderated mediation: low ISI indirect	−0.093	[−0.208, 0.006]	—
T1 moderated mediation: mean ISI indirect	−0.024	[−0.088, 0.036]	—
T1 moderated mediation: high ISI indirect	0.044	[−0.012, 0.109]	—
T1 moderated mediation: index	0.069	[0.013, 0.137]	—
T2 moderated mediation: GAISV × ISI → CM	0.361	[0.086, 0.631]	—
T2 moderated mediation: low ISI indirect	−0.037	[−0.118, 0.006]	—
T2 moderated mediation: mean ISI indirect	−0.007	[−0.043, 0.022]	—
T2 moderated mediation: high ISI indirect	0.024	[−0.003, 0.078]	—
T2 moderated mediation: index	0.031	[−0.001, 0.087]	—

CM, cognitive mirroring; ISI, image self-identification; SCC, self-concept clarity. ANCOVA models controlled for baseline SCC, self-esteem, trait self-reflection, AI experience, and age. Mediation and moderated mediation estimates are unstandardized coefficients with bootstrap 95% confidence intervals. The T1 moderated mediation index is the primary test of H4. Conditional indirect effects are reported to show the pattern across ISI levels; the individual conditional confidence intervals are close to or include zero and should not be interpreted as separate significant conditional effects. The T2 models are short-term follow-up robustness analyses. In all mediation and moderated mediation models, condition was coded GAISV = 1 versus the two image-control conditions combined = 0, every equation included the five ANCOVA covariates, and ISI was standardized. Relative indirect effects for each control condition and results with self-description only as the baseline are reported in Supplementary materials S14, S15.

### 3.3 Mediation and competing mediation

H2 and H3 were tested in parallel mediation models with cognitive mirroring and flow as simultaneous mediators. At T1, GAISV (relative to the two image-control conditions combined) positively predicted cognitive mirroring, B = 0.494, 95% CI [0.302, 0.692], which in turn predicted T1 SCC, B = 0.226, 95% CI [0.155, 0.299]. The indirect effect through cognitive mirroring was positive, B = 0.112, 95% CI [0.062, 0.173], supporting H2.

In the same model, GAISV also predicted flow, B = 0.258, 95% CI [0.041, 0.499], but flow did not reliably predict T1 SCC once cognitive mirroring was included, B = 0.038, 95% CI [−0.031, 0.111]. The flow indirect effect was small, with a confidence interval including zero, B = 0.010, 95% CI [−0.007, 0.038], and the direct effect of GAISV was B = 0.094, 95% CI [−0.016, 0.210]. The contrast between indirect effects favored cognitive mirroring, B = 0.102, 95% CI [0.044, 0.168], consistent with H3. The same pattern held when GAISV was compared with each control condition separately: the relative indirect effect through cognitive mirroring was B = 0.117, 95% CI [0.061, 0.188], versus passive viewing and B = 0.106, 95% CI [0.053, 0.174], versus active non-self, whereas the relative indirect effect through flow was unreliable in both comparisons (Supplementary material S14).

A T2 parallel mediation model served as a follow-up robustness check. GAISV predicted cognitive mirroring, B = 0.526, 95% CI [0.324, 0.725], which predicted T2 SCC, B = 0.088, 95% CI [0.004, 0.168], giving a positive cognitive-mirroring indirect effect, B = 0.046, 95% CI [0.002, 0.096]. GAISV again predicted flow, B = 0.290, 95% CI [0.054, 0.530], but flow did not reliably predict T2 SCC, B = 0.027, 95% CI [−0.054, 0.108], and its indirect effect was B = 0.008, 95% CI [−0.015, 0.040]. The T2 indirect contrast was weaker than at T1 and included zero, B = 0.038, 95% CI [−0.017, 0.094], and the direct effect was B = 0.175, 95% CI [0.036, 0.307]. T1 and T2 estimates appear in Table 3. Relative to each control condition separately, the T2 indirect effect through cognitive mirroring was B = 0.050, 95% CI [0.003, 0.108] (passive viewing) and B = 0.043, 95% CI [0.002, 0.091] (active non-self).

### 3.4 Moderated mediation and robustness checks

H4 tested whether image self-identification moderated the indirect path from GAISV to SCC through cognitive mirroring. In the T1 model, the GAISV × image self-identification interaction on cognitive mirroring was positive, B = 0.338, 95% CI [0.072, 0.597], and the index of moderated mediation was positive with a confidence interval excluding zero, B = 0.069, 95% CI [0.013, 0.137], supporting H4 at the post-task assessment.

Conditional indirect effects were estimated at low, mean, and high image self-identification: B = −0.093, 95% CI [−0.208, 0.006] (low); B = −0.024, 95% CI [−0.088, 0.036] (mean); and B = 0.044, 95% CI [−0.012, 0.109] (high). The point estimates rose across levels, but each individual conditional interval was close to or included zero, so support for H4 rests on the moderated-mediation index rather than on any single conditional effect. Figure 3 displays the conditional indirect effects, and full estimates appear in Table 3. Decomposed by control condition, the T1 index was reliable relative to passive viewing, B = 0.085, 95% CI [0.020, 0.168], but its confidence interval included zero relative to active non-self, B = 0.052, 95% CI [−0.005, 0.124] (Supplementary material S14); the post-task moderation is therefore clearest in the comparison with passive viewing.

**Figure 3:**
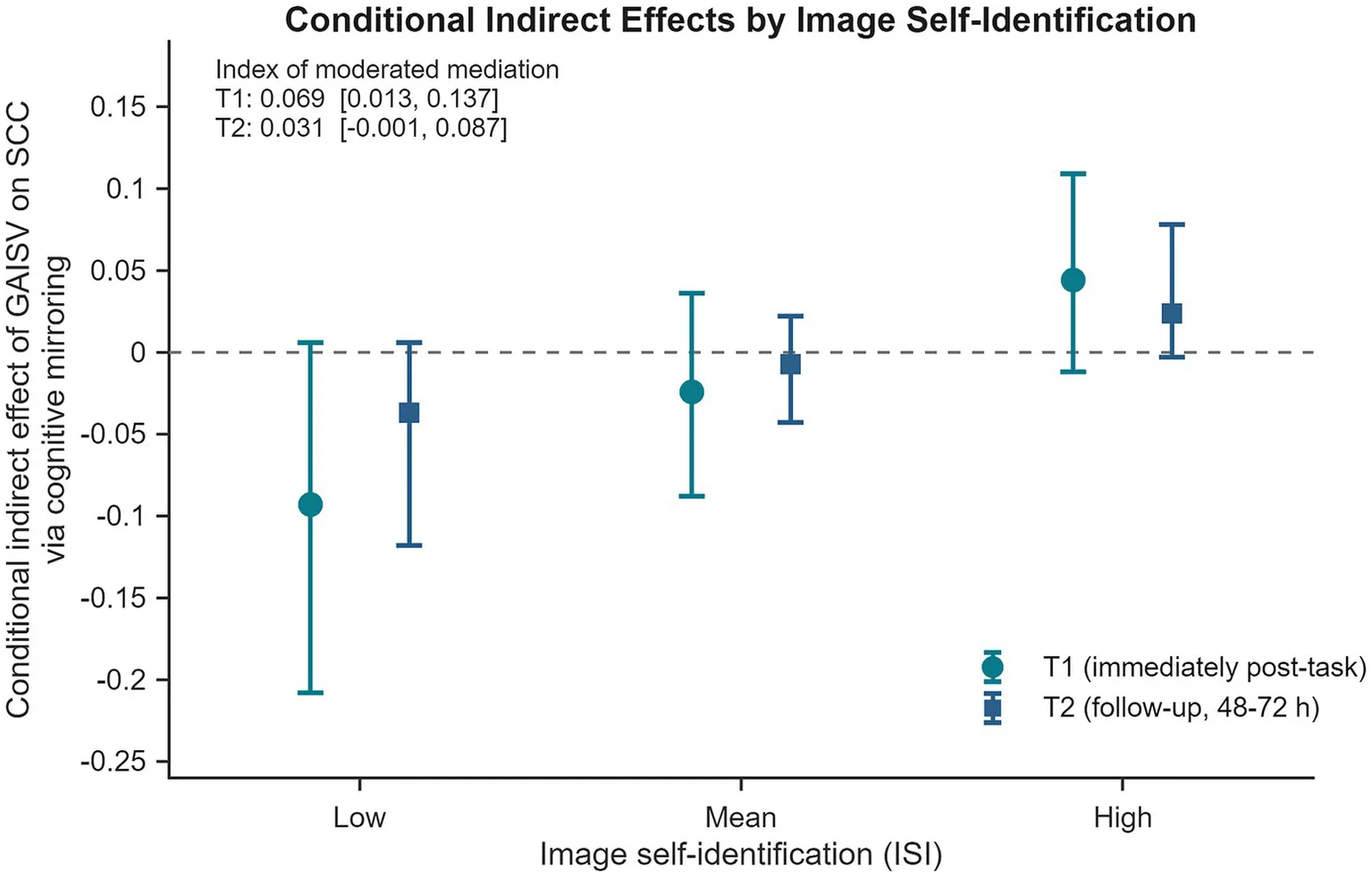
Conditional indirect effects of GAISV on T1 SCC through cognitive mirroring across levels of image self-identification (low = −1 SD, mean, high = +1 SD). Point estimates increase across levels, but the individual conditional confidence intervals are close to or include zero; the figure should be read together with the significant T1 index of moderated mediation rather than as evidence that each conditional indirect effect is independently significant.

A T2 moderated mediation model served as a robustness check. The GAISV × image self-identification interaction on cognitive mirroring remained positive, B = 0.361, 95% CI [0.086, 0.631]. Conditional indirect effects were B = −0.037, 95% CI [−0.118, 0.006] (low); B = −0.007, 95% CI [−0.043, 0.022] (mean); and B = 0.024, 95% CI [−0.003, 0.078] (high). The T2 index was B = 0.031, 95% CI [−0.001, 0.087]—at the zero boundary—so the follow-up did not support H4 as clearly as the post-task model.

Additional checks evaluated item and response quality. Item-loading diagnostics were within acceptable ranges, with expected variation across items and reverse-scored items. Respondent-style indicators flagged 30 cases with at least one straight-line scale pattern in the primary analysis sample; these flags were kept for diagnostics and did not change the effective sample after the pre-specified exclusions. Subgroup diagnostics by image self-identification tertiles were retained as supplementary checks. Brief T2 recall items (image memory and spontaneous self-reflection) and the T1 safety-monitoring items were collected for descriptive purposes only and are summarized in the Supplementary material; they were not part of the primary H1–H4 tests. Together these support the use of the primary sample and appear in the Supplementary material.

Results were also robust to image-level variation: image quality (MC3) and perceived realism (MC4) did not differ across conditions, *F*(2, 243) = 0.25, *p* = 0.783 and *F*(2, 243) = 0.43, *p* = 0.651, and re-estimating the primary models with these two variables as covariates left the GAISV contrasts essentially unchanged (Supplementary material S3b).

Exploratory comparison with self-description only. As a preplanned exploratory contrast using the same covariates, GAISV showed a small advantage over the self-description-only condition in post-task SCC, B = 0.146, 95% CI [0.002, 0.290], *p* = 0.047, and an estimate of similar size at follow-up whose confidence interval included zero, B = 0.164, 95% CI [−0.003, 0.331], *p* = 0.054. The AI-generated self-image thus contributed a modest increment beyond self-description alone—smaller than the GAISV advantage over the passive- and active-control conditions—and this comparison is interpreted as exploratory.

Mediation with self-description only as the baseline. Because GAISV and the self-description-only condition both involved writing about the self, we repeated the parallel mediation model comparing GAISV (*n* = 80) with self-description only (*n* = 82) as an additional exploratory analysis, using the same covariates. At T1, GAISV predicted higher cognitive mirroring than self-description only, B = 0.254, 95% CI [0.021, 0.475], and the indirect effect through cognitive mirroring was positive but smaller than in the primary model, B = 0.068, 95% CI [0.005, 0.137]; the indirect effect through flow was not reliable, B = 0.003, 95% CI [−0.016, 0.026]. At T2 (n = 76 and 70), the indirect effect through cognitive mirroring was not reliable, B = 0.025, 95% CI [−0.005, 0.069]. In a four-group model with self-description only as the reference, cognitive mirroring was also lower after passive viewing than after self-description only, B = −0.267, 95% CI [−0.487, −0.043], and directionally lower after active non-self, B = −0.202, 95% CI [−0.408, 0.009], suggesting that writing about the self engaged some reflective processing on its own and that the AI-generated self-image added a further, more modest increment. The moderated mediation model could not be estimated with self-description only as the baseline because image self-identification was not measured in that condition. Full estimates appear in Supplementary material S15.

Differential attrition. Follow-up retention in the T2 analysis sample was 95.0% (GAISV), 83.3% (active non-self), and 85.4% (passive viewing); the difference across conditions was not statistically significant, χ^2^(2) = 5.89, *p* = 0.053. Participants retained at T2 did not differ from those not retained on baseline SCC, post-task SCC, cognitive mirroring, flow, image self-identification, self-esteem, trait self-reflection, age, or AI experience (all *p* > 0.10). Attrition was therefore unrelated to the measured baseline and post-task variables, making it an unlikely source of bias in the T2 estimates. As a sensitivity analysis, we re-estimated the T2 condition effects under multiple imputation (m = 30, recovering the full N = 246); the GAISV advantages were essentially unchanged and remained significant (vs. passive viewing B = 0.19, 95% CI [0.05, 0.34], *p* = 0.010; vs. active non-self B = 0.21, 95% CI [0.06, 0.35], *p* = 0.006), matching the complete-case estimates.

## 4 Discussion

### 4.1 Principal findings and theoretical contributions

This study asked whether generative AI-assisted self-visualization raises self-concept clarity through reflective cognitive mirroring rather than through general immersive flow. Across a pilot and a randomized experiment, the results matched the mechanism-adjudication account. GAISV produced higher post-task and follow-up SCC than both the active non-self and passive viewing controls. In the parallel mediation model, the effect was statistically consistent with a cognitive-mirroring pathway, whereas the flow path was weak and unreliable, and the indirect contrast favored cognitive mirroring immediately after the task. The value of GAISV thus lies less in making an AI task engaging and more in letting participants use an AI-generated self-image as a reflective object for organizing self-understanding. Because the mediators and the post-task outcome were assessed in the same session, these results are mechanism-consistent statistical associations and do not establish causal ordering among the post-task states. Relative to writing a self-description alone, however, the AI-generated self-image added a smaller increment in cognitive mirroring at the post-task assessment and no reliable increment at follow-up, suggesting that part of the reflective effect comes from describing the self.

The main contribution is not a broad claim that AI self-images are beneficial, but the adjudication between two plausible mechanisms within one experimental model. Flow rose with the image task, confirming that AI image generation and viewing can be engaging—yet that immersion did not account for SCC change once cognitive mirroring was modeled alongside it. This matters because much of the appeal of generative AI comes from novelty, visual richness, and absorption, and the present findings show these features are not enough to explain SCC change. The data instead point to a specific reflective mechanism: participants benefited when the image helped them observe and organize self-relevant material.

The magnitude of these effects should be read with care. The condition contrasts corresponded to differences of roughly 0.19–0.24 SCC scale points and to small standardized effects (Cohen’s d ≈ 0.21–0.27; partial η^2^ ≈ 0.05–0.06). Because SCC is a comparatively stable self-structure, a brief task would not be expected to move it substantially; the present results are best described as a small, short-term shift in perceived clarity rather than evidence that GAISV constitutes a substantial self-development intervention.

The findings also give initial support to the AI Mirror Self-Integration Model, which links self-externalization, AI-mediated visual feedback, cognitive mirroring, image self-identification, and SCC in one account of human–AI interaction. We frame this as a structure for organizing testable mechanisms, not a finished theory. The pilot validation of CM-HAIS and the experimental results together suggest that cognitive mirroring can be operationalized as a state-like process in AI self-visualization, though further validation across platforms and populations is needed before it can be treated as a mature construct. We emphasize that only the condition-to-SCC path was identified by random assignment; cognitive mirroring and image self-identification were measured after the task and therefore provide correlational process evidence rather than experimentally identified paths (Figure 1).

Image self-identification refined the picture. Image self-identification was statistically associated with the strength of the cognitive-mirroring path at the post-task assessment, so AI self-images were more likely to act as meaningful mirrors when participants saw them as personally identifying; when identification was weak, an image may stay a visual artifact rather than a reflective self-object. The T2 results warrant more caution: the cognitive-mirroring path remained clearer than the flow path at follow-up, but the moderated mediation evidence weakened. Because image self-identification was measured after the manipulation and was itself elevated by GAISV, it is best understood as a post-task conditional process variable rather than an exogenous, causal boundary condition; experimentally manipulating image congruence or self-identification is needed to test moderation directly.

### 4.2 Relation to prior work

The results fit foundational and recent work on SCC as an index of self-structure and identity integration. Campbell et al. (1996) framed SCC as the clarity, consistency, and stability of self-beliefs, and later work tied it to self-expansion (Emery et al., 2022), temporal self-comparison (Zaw and Baldwin, 2023), and computational accounts of self-structure (Garcia-Arch et al., 2025). Newer SCC studies in clinical and student populations also support its role as an integration-related resource rather than a narrow self-esteem proxy (Antichi et al., 2026; Dong et al., 2026; Kallai et al., 2026). We extend this to a new self-representational context in which participants met an AI-mediated visualization of their own self-description, and we find that such externalized representations matter for SCC mainly when they support reflective organization rather than simply offering new or attractive self-content.

Evidence on digital media and the self has been mixed. Digital environments can support identity exploration but can also bring fragmented self-presentation (Rafiq and Linden, 2024; Soh et al., 2024) and, longitudinally, digital stress that tracks lower self-concept clarity (Dodan and Negru-Subtirica, 2025), and short-video research has linked flow and self-expansion to SCC (Wang and Wang, 2024). Our results partly converge—visual AI tasks did raise immersion—but diverge in that flow did not explain SCC change under GAISV. Not all digital engagement relates to self-clarity in the same way; here the key distinction is between being absorbed in the task and using the AI output to reflect on the self.

Research on algorithmic selfhood and human-AI interaction offers a further point of contact. Prior work holds that algorithmic platforms shape self-making on social media (Bhandari and Bimo, 2022), that users actively legitimate algorithmic sensemaking (Obreja, 2024), and that AI more broadly reshapes identity and introspection (Joseph, 2025). Classic work also shows that people may respond socially to computational agents and attribute agency or meaning to them under some conditions (Nass and Moon, 2000; Epley et al., 2007; Waytz et al., 2010). Trust research extends this point to contemporary AI systems (Glikson and Woolley, 2020). Studies of AI companions and human-AI relationships similarly show that relational framing, attachment, and privacy shape how people interpret artificial agents (Chaturvedi et al., 2023; Register et al., 2025; Skjuve et al., 2021). We move from broad claims about AI reshaping identity to a tested mechanism: GAISV did not merely expose participants to an algorithmic output but turned their own self-description into a visual object, which functioned as an externalized self-representation only when engaged through cognitive mirroring.

They are also compatible with cautionary work on AI-generated images, which can be realistic, persuasive, biased, or psychologically consequential (Miller et al., 2023; Miskolczi, 2026). Body-image and eating-disorder research further warns that AI-generated visual content may heighten self-discrepancy or appearance-related concerns in vulnerable contexts (Higgins, 1987; Bennett et al., 2026; Craddock et al., 2026; Weinmann et al., 2026). Realistic AI-generated images may also affect public judgment when their artificial origin becomes salient (Bunzli and Eppler, 2026; Liu et al., 2026). Our results do not contradict these concerns; they help explain why AI self-images are not uniformly positive self-development tools. Their effects depend on whether the image is experienced as personally identifying and whether it supports reflective integration-an account compatible with both the promise and the risks of generative AI in self-related applications.

### 4.3 Practical and design implications

The results have implications for designing AI self-visualization tools. Because these design features were not manipulated in the present study, the following are offered as design hypotheses for future testing rather than as established prescriptions. To support self-understanding, designers should not optimize only for realism, novelty, or immersion: flow rose without explaining SCC change. Tools meant for self-reflection should instead foster cognitive mirroring, helping users examine how their self-description relates to the generated output, notice self-relevant patterns, and integrate scattered self-feelings into a more coherent whole.

One implication is transparency between input and output. Users may benefit from seeing how parts of their self-description became visual features, and from comparing the image with their original text. Human-AI design work also recommends communicating system capabilities and limits so that users can calibrate trust and agency during collaboration (Jiang et al., 2024), while broader reviews specify when combining human and AI judgment actually helps (Vaccaro et al., 2024) and stress ethical responsibility in such collaboration (Canas, 2022). Reflection prompts-asking what feels accurate, surprising, incomplete, or inconsistent-could shift the experience from passive viewing toward reflective processing and help separate meaningful self-visualization from entertainment.

A second implication concerns user control. Because image self-identification may act as a post-task conditional factor rather than an established boundary condition, systems could allow users to adjust, regenerate, reject, or annotate AI images. A low-identification image may not support cognitive mirroring and could even produce alienation or self-discrepancy, so systems should offer re-generation, explanation, or deletion, especially when users report that an image does not represent them. Work on artificial companions likewise suggests that continuity, relational cues, and attachment can shape user experience in ways that require explicit safeguards (Laestadius et al., 2022; Liu et al., 2025; Rogge, 2023). This matters most in sensitive settings-education, counseling, digital health, and identity-development interventions-where AI images can carry meaning beyond their visual form.

Finally, the study favors a cautious application model. GAISV may help as a brief reflective aid, but it should not be presented as a standalone intervention or a substitute for professional support. Its most defensible use is as a structured self-reflection tool that pairs AI output with user agency, transparent prompts, privacy protection, and safeguards for discomfort or self-discrepancy. In short, generative AI self-visualization should be designed to clarify, not merely immerse, the self.

### 4.4 Limitations and future directions

Several limitations qualify these conclusions. First, the study used an online behavioral experiment in one language and sample context, so generalizability to other cultural, linguistic, developmental, or clinical populations is untested. SCC and image self-identification may work differently where self-construal, visual self-presentation, or attitudes toward AI differ. Replication in more diverse samples, and tests of whether the cognitive-mirroring path varies across contexts, would help. In particular, because eligibility required recent generative-AI use (at least once in the past 30 days), the sample comprised adults with current AI experience and does not represent people with no generative-AI exposure.

Second, the AI procedure used standardized prompts. This improved control and reduced privacy risk by avoiding face uploads, but it may also have limited image self-identification. In addition, the underlying web-based generation model was not version-locked across the collection window, so the specific model could have changed over time even though the prompt template and procedure were fixed. Other forms of AI self-visualization—editable avatars, future-self images, interactive refinement, or multimodal dialogue—might produce stronger or weaker cognitive mirroring, and comparing models, prompt structures, and degrees of user control would clarify which features promote reflective integration without raising self-discrepancy or discomfort. Because model version and generation date/time were not systematically recorded, a version- or date-based robustness check was not feasible. As an alternative using only recorded data, the image features entering analysis were comparable across conditions—image quality and perceived realism did not differ between GAISV and the controls—and adding these image-level variables as covariates did not alter the GAISV effect on SCC (Supplementary material S3b). This indicates that any cross-version drift did not operate through the measured image characteristics, although fully controlled tests will require version-locked generation (e.g., a fixed API model) in future work.

Third, although this study adds confirmatory discriminant-validity evidence (two-factor CFA, average variance extracted, and HTMT) indicating that cognitive mirroring is empirically separable from SCC, trait self-reflection, private self-consciousness, and rumination, CM-HAIS was developed here and still needs further validation. The pilot showed acceptable internal consistency and preliminary separation from adjacent constructs, but cognitive mirroring remains conceptually close to self-reflection, private self-consciousness, and self-observation. Future work should test its factor structure in larger independent samples, compare it with established reflection and rumination measures, and check whether it predicts outcomes beyond SCC; experimental manipulations of cognitive mirroring would strengthen causal claims.

Fourth, the outcome relied on self-reported SCC measured shortly after the task and at a brief follow-up, which suits immediate and short-term processes but does not establish durable change. As noted, the T1 mediators and T1 SCC came from the same session, so—despite randomization and the T2 follow-up—the mediation models are mechanism-consistent rather than proof of causal ordering. Longer follow-ups, diary assessments, behavioral indicators, interviews, or designs that temporally separate mediator and outcome would help establish whether GAISV produces lasting integration or mainly a temporary rise in perceived clarity.

Fifth, the moderated mediation findings call for restraint. The post-task index was consistent with image self-identification qualifying the cognitive-mirroring path, but the conditional indirect effects were modest, the T2 evidence was weaker, and when the two image controls were examined separately the index was reliable only relative to passive viewing. Image self-identification was also measured after the manipulation and was much higher after GAISV than after either image control (M = 4.86 vs. 3.09 and 3.15), so it may itself have been shaped by the condition and cannot be treated as a pre-existing moderator. In addition, the moderated mediation model could not be estimated with self-description only as the baseline because that condition involved no image. Future studies could manipulate it directly—varying prompt personalization, allowing image revision, or comparing high- and low-congruence outputs—to test this moderation directly.

Finally, the self-description-only condition was an exploratory comparison rather than part of the primary model. Because the CM-HAIS was developed for responses to an AI image, cognitive-mirroring scores in this condition, which involved no image, should be compared with those in the image conditions with caution. Future work should use a fully crossed design to separate the effects of self-description, AI generation, image viewing, and self-identification, and to test whether SCC change comes from writing about the self, seeing the image, reflecting on it, or their interaction. Such work should also examine risks, including self-discrepancy, biased generation, privacy, and the possibility that AI images distort rather than clarify self-understanding for some users.

## 5 Conclusion

This study proposed and tested the AI Mirror Self-Integration Model to explain how generative AI-assisted self-visualization relates to self-concept clarity in human–AI interaction. GAISV raised short-term SCC, but not because AI self-images are inherently beneficial. The effect was statistically consistent with a cognitive-mirroring process: participants gained when the image supported reflective self-observation and the organization of self-relevant material, whereas flow rose with the task but did not explain the gain. This reflective-over-immersive advantage was clearest immediately after the task and weakened at the 48–72 h follow-up. The condition differences were small in magnitude (Cohen’s d ≈ 0.21–0.27) and should be read as a modest, short-term shift in perceived clarity rather than a substantial self-development intervention.

Image self-identification further qualified this process, with AI self-images more likely to support the cognitive-mirroring path when participants experienced them as personally identifying. Generative AI self-visualization is therefore better understood not as a universally clarifying technology but as a context-dependent reflective tool—one that can help organize the self when its images feel personally identifying and prompt reflection rather than mere absorption.

## Data Availability

The original contributions presented in the study are included in the article/Supplementary material, further inquiries can be directed to the corresponding author.
