# Supplementary material for: Generative AI-assisted self-visualization and self-concept clarity: evidence consistent with a cognitive-mirroring mechanism in human–AI interaction
Source: Front Psychol. 2026 Oct 28;17:1918225. doi: 10.3389/fpsyg.2026.1918225 (PMC13752073; doi:10.3389/fpsyg.2026.1918225)
Supplement: Supplementary file 1 [file Supplementary_file_1.DOCX]

Supplementary Material

# S1. Study Design and Sample Flow

The study used a two-stage design. The pilot study contained 132 responses, of which 124 passed the attention-check and data-quality criteria and were retained for pilot validation of the Cognitive Mirroring in Human-AI Self-Visualization Scale (CM-HAIS).

The main experiment contained 376 raw platform records and 328 valid participants after pre-specified exclusions. The primary H1-H4 analyses used three conditions: generative AI-assisted self-visualization (GAISV; n = 80), active non-self image generation (n = 84), and passive image viewing (n = 82), yielding N = 246. The self-description-only group (n = 82) is retained as an exploratory supplementary comparison and is not part of the primary H1-H4 models.

At the 48-72 h follow-up, 221 participants in the three primary-analysis groups completed T2, and 216 met the pre-specified T2 analysis criteria after follow-up quality checks.

# S2. Measures and Variable Mapping

All attitudinal items used 7-point Likert-type response scales unless otherwise specified. Scale scores were computed as item means after reverse scoring where applicable. Reverse-scored items were SCC3, SCC5, SCC8, SCC10 at each SCC time point; CM6; ISI5; RSE3; and SR4.

The file main_experiment_variable_dictionary_expanded.csv provides the machine-readable variable dictionary, including construct labels, coding rules, reverse-scoring information, analysis role, and missing-value notes.

# S3. AI Image-Generation Procedure and Privacy Safeguards

AI images were produced with OpenAI's image-generation feature in ChatGPT ("ChatGPT Images") via the ChatGPT web interface between 23 June 2025 and 13 March 2026. Because the web interface did not expose a fixed model version, visible model/version records were retained where available at the generation-session level rather than treated as a fixed experimental constant.

The task procedure was standardized across participants. In the GAISV condition, participants wrote an 80-150 word self-description and viewed one AI-generated self-representation image. In the active non-self condition, participants wrote an 80-150 word description of a fictional or neutral non-self target and viewed one AI-generated non-self image. In the passive viewing condition, participants viewed a pre-generated, style-matched neutral image. In the self-description-only condition, participants wrote a self-description but did not generate or view an AI image.

Participants did not upload real face photographs and were not asked for real names, school or company affiliations, addresses, or contact details. Raw participant-generated prompts, reflective free text, and AI-generated participant images are not included in the public supplementary data package because they may contain identifiable or sensitive self-related material.

# S4. Pilot CM-HAIS Validation

In the pilot valid sample (n = 124), the 7-item CM-HAIS showed acceptable internal consistency (Cronbach's alpha = 0.846). A one-factor extraction gave a first eigenvalue of 3.703, with item loadings ranging from 0.579 to 0.808. The pilot outputs are provided in Pilot_CM_HAIS_validation.csv, Pilot_CM_HAIS_condition_descriptives.csv, and Pilot_CM_HAIS_validity_correlations.csv.

# S5. Manipulation Checks, T2 Retention, and Diagnostics

Participant characteristics and focal manipulation-check variables are provided in Table1_participant_characteristics_and_checks_by_condition.csv. T2 retention and analysis eligibility are documented in T2_retention_and_analysis_audit.csv. Additional manipulation-check contrasts, item-loading diagnostics, respondent-style diagnostics, and heterogeneous subgroup diagnostics are provided as machine-readable CSV files.

# S6. Statistical Outputs and Figure Data

The main manuscript tables are supported by Table1_participant_characteristics_and_checks_by_condition.csv, Table2_descriptives_reliability_correlations_descriptives.csv, Table2_descriptives_reliability_correlations_alpha_loadings.csv, Table2_descriptives_reliability_correlations_matrix.csv, and Table3_effects_mediation_moderated_mediation.csv.

Figure 3 is supported by Figure3_conditional_indirect_effects_data.csv. PNG source images for Figures 1-3 are included in the figures folder of the supplementary data ZIP; the publication-quality figures are uploaded separately as high-resolution TIFF.

# S7. Public Supplementary Data and Controlled-Access Materials

The accompanying file Supplementary_Data_Tables_Figures_20260610.zip contains machine-readable supplementary tables, outputs, figure data, vector figures, a manifest, and two de-identified scale-level datasets. The de-identified main-experiment scale file removes original participant IDs, timestamps, occupation, country/region, image IDs, model-version records, raw prompt text, and free-text reflection fields.

The raw N376 platform export, the full N328 effective-sample file with prompt text, participant-generated self-descriptions, reflective free text, and AI-generated participant images are not publicly released. These materials are available only under controlled conditions where consistent with the ethics approval and the Data Availability Statement.

# S8. Supplementary Data File Checklist

Key files in the public ZIP include: S_Data_main_scale_level_deidentified_N328.csv; S_Data_pilot_CM_HAIS_deidentified_N132.csv; Table1-Table3 CSV source files; T2_retention_and_analysis_audit.csv; Figure3_conditional_indirect_effects_data.csv; manipulation-check, reliability, respondent-style, and subgroup diagnostic CSV files; the expanded variable dictionary; Figure 1-3 PNG source images; README_Supplementary_Data_20260610.md; and Supplementary_Data_Manifest_20260610.csv.

***— Additional analyses added in revision (in response to Reviewers) —***

**S9. Participant Flow and Exclusions (Revision)**

The main-experiment raw platform export contained 376 records; after the pre-specified exclusion rules (missing consent, failed eligibility screen, failed attention checks, incomplete main-session data, missing key variables, task non-compliance, extreme completion time, duplicate/invalid records, and failed or unusable image generation where required), 328 participants were retained. The same rules were applied uniformly across conditions. Random assignment occurred after the eligibility screen and consent; the small number of post-assignment exclusions (e.g., image-generation failure) were handled by the same pre-specified rules in each condition. Per-case exclusion reasons were not retained as fields in the de-identified analysis dataset, so exclusions are summarized at the criterion level overall rather than cross-tabulated by condition and criterion. Follow-up (T2) retention by condition is shown below.

| **Condition** | **T1 (n)** | **Completed T2** | **Retained in T2 analysis** | **T2 retention (%)** |
| --- | --- | --- | --- | --- |
| GAISV | 80 | 77 | 76 | 95.0 |
| Active non-self | 84 | 73 | 70 | 83.3 |
| Passive viewing | 82 | 71 | 70 | 85.4 |
| Total (primary analysis) | 246 | 221 | 216 | 87.8 |

*Note. Retention did not differ significantly across conditions, chi-square(2) = 5.89, p = 0.053 (see S11).*

**S10. Discriminant and Convergent Validity of CM-HAIS (Revision)**

To address the concern that cognitive mirroring (CM-HAIS) might overlap with self-concept clarity (SCC), we compared a two-factor model (CM vs. post-task SCC) with a one-factor model in the primary analysis sample (N = 246), using maximum-likelihood confirmatory factor analysis on standardized items.

| **Model** | **chi-square** | **df** | **p** | **CFI** | **TLI** | **RMSEA** | **SRMR** |
| --- | --- | --- | --- | --- | --- | --- | --- |
| One-factor (CM + SCC combined) | 768.1 | 152 | <.001 | 0.758 | 0.727 | 0.129 | 0.140 |
| Two-factor (CM / SCC separate) | 159.6 | 151 | .300 | 0.997 | 0.996 | 0.015 | 0.035 |

The two-factor model fit well and far better than the one-factor model (delta chi-square(1) = 608.5); for the two-factor model chi-square was close to its degrees of freedom (159.6 vs. 151, p = .300), indicating good fit rather than perfect fit. The latent CM-SCC correlation was moderate (r = 0.388). Convergent/discriminant indices: AVE(CM) = 0.519, CR(CM) = 0.882; AVE(SCC) = 0.555, CR(SCC) = 0.936; the square roots of AVE (0.720 and 0.745) exceeded the inter-construct correlation (Fornell-Larcker criterion). The heterotrait-monotrait ratio (HTMT = 0.398) is our primary discriminant index because it is computed directly from the item correlations and is independent of model estimation; it was well below the 0.85 threshold.

In the pilot sample (n = 124 retained), cognitive mirroring was likewise distinguishable from adjacent constructs:

| **Comparison** | **Latent r** | **HTMT** | **chi-square(df)** | **p** | **CFI** | **RMSEA** | **SRMR** |
| --- | --- | --- | --- | --- | --- | --- | --- |
| CM vs. trait self-reflection | 0.290 | 0.296 | 29.6 (43) | .941 | 1.000 | 0.000 | 0.041 |
| CM vs. private self-consciousness | 0.432 | 0.435 | 33.3 (43) | .856 | 1.000 | 0.000 | 0.037 |
| CM vs. rumination | 0.223 | 0.241 | 61.6 (43) | .033 | 0.965 | 0.059 | 0.054 |

For the two smaller pilot models, chi-square fell below the degrees of freedom (hence RMSEA = 0.000); this reflects the parsimony of these 11-item, single-respondent models rather than overfitting, and the CM vs. rumination model showed non-saturated fit (RMSEA = 0.059, p = .033). Across all comparisons HTMT was well below 0.85 and Fornell-Larcker held, indicating that cognitive mirroring is related to but empirically distinct from SCC, trait self-reflection, private self-consciousness, and rumination. All CFA and HTMT results were produced by analysis scripts that reproduce the reported values exactly and are available on request.

**S11. Differential Attrition Analysis (Revision)**

Retention across the three primary-analysis conditions did not differ significantly, chi-square(2) = 5.89, p = 0.053. Participants retained at T2 were compared with those not retained on baseline and post-task variables (Welch t-tests); none differed significantly, consistent with a missing-at-random pattern.

| **Variable** | **Retained (M)** | **Not retained (M)** | **t** | **p** |
| --- | --- | --- | --- | --- |
| Baseline SCC (T0) | 4.63 | 4.58 | 0.29 | .774 |
| Post-task SCC (T1) | 4.67 | 4.68 | -0.02 | .981 |
| Cognitive mirroring | 4.21 | 4.10 | 0.75 | .458 |
| Flow | 4.25 | 4.33 | -0.48 | .636 |
| Image self-identification | 3.72 | 3.43 | 1.56 | .127 |
| Self-esteem | 4.80 | 4.72 | 0.63 | .535 |
| Trait self-reflection | 4.58 | 4.71 | -1.00 | .323 |
| Age | 31.71 | 32.27 | -0.29 | .773 |
| AI experience | 4.53 | 4.80 | -1.65 | .107 |

As a sensitivity analysis, the follow-up (T2) condition effects were re-estimated under multiple imputation (m = 30, recovering the full N = 246, imputing T2 SCC from baseline and T1 measures, covariates, and condition). The GAISV advantages were essentially unchanged and remained significant (GAISV vs. passive viewing B = 0.19, 95% CI [0.05, 0.34], p = 0.010; GAISV vs. active non-self B = 0.21, 95% CI [0.06, 0.35], p = 0.006), closely matching the complete-case estimates, indicating that differential attrition is an unlikely source of bias in the T2 results.

**S12. Exploratory Comparison: GAISV vs. Self-Description-Only (Revision)**

As a preplanned exploratory contrast (ANCOVA with baseline SCC, self-esteem, trait self-reflection, AI experience, and age as covariates), GAISV was compared with the self-description-only condition.

| **Outcome** | **N (GAISV / self-desc)** | **Adjusted difference B** | **95% CI** | **p** |
| --- | --- | --- | --- | --- |
| Post-task SCC (T1) | 162 (80 / 82) | 0.146 | [0.002, 0.290] | .047 |
| Follow-up SCC (T2) | 146 (76 / 70) | 0.164 | [-0.003, 0.331] | .054 |

The AI-generated self-image contributed a modest increment beyond self-description alone at T1; at follow-up the estimate was of similar size but its confidence interval included zero. Both estimates were smaller than the GAISV advantage over the passive- and active-control conditions; this comparison is interpreted as exploratory.

**S13. Statistical-Reporting Correction (Revision)**

All reported values were regenerated directly from the analysis scripts operating on the de-identified data. Three ANCOVA pairwise p-values previously shown as p < .001 are corrected to their exact values: T1 GAISV vs. active non-self, p = .008; T2 GAISV vs. passive viewing, p = .003; T2 GAISV vs. active non-self, p = .002 (T1 GAISV vs. passive viewing remains p < .001). All coefficients, standard errors, and confidence intervals reproduced exactly; the corrections do not change any conclusion. In the second revision, one further transcription error was corrected: the p value for the T1 contrast between the active non-self and passive-viewing conditions is .483 rather than .474 (B and 95% CI unchanged; non-significant either way). In Table S3b, the p value for the T1 primary GAISV vs. passive-viewing contrast is shown as < .001, consistent with Table 3.

**S3b. Robustness to Image-Level Variation (Revision)**

Because the generation model version and generation date/time were not systematically recorded, a version- or date-based robustness check was not feasible. We instead assessed whether the image characteristics entering analysis were comparable across conditions and whether the condition effect was sensitive to them. (i) Comparability: image quality and perceived realism did not differ across GAISV, active non-self, and passive viewing (Table S3a). (ii) Insensitivity: adding image quality (MC3) and perceived realism (MC4) as covariates left the GAISV contrasts essentially unchanged (Table S3b); results were equivalent using the multi-item image-quality composite (F(2, 243) = 1.02, p = .363). Image personalization and self-similarity differed by design (they reflect the self-relevance manipulation: e.g., personalization F(2, 243) = 240.85, p < .001) and are therefore NOT used as standardization covariates, since doing so would partial out the treatment itself.

*Table S3a. Image technical features by condition, M (SD).*

| **Variable** | **GAISV** | **Active non-self** | **Passive viewing** | **F(2, 243)** | **p** |
| --- | --- | --- | --- | --- | --- |
| Image quality (MC3) | 5.00 (0.95) | 4.93 (1.02) | 5.04 (1.06) | 0.25 | .783 |
| Perceived realism (MC4) | 4.50 (1.20) | 4.64 (1.00) | 4.62 (0.96) | 0.43 | .651 |

*Table S3b. GAISV contrasts on SCC before vs. after adjusting for image quality and realism.*

| **Model** | **GAISV - passive: B [95% CI], p** | **GAISV - active: B [95% CI], p** |
| --- | --- | --- |
| T1 primary | 0.241 [0.100, 0.382], < .001 | 0.191 [0.050, 0.332], .008 |
| T1 + quality & realism | 0.245 [0.103, 0.387], .001 | 0.198 [0.056, 0.340], .007 |
| T2 primary | 0.227 [0.079, 0.374], .003 | 0.232 [0.085, 0.380], .002 |
| T2 + quality & realism | 0.231 [0.082, 0.379], .002 | 0.236 [0.087, 0.385], .002 |

***— Additional analyses added in the second revision (in response to Reviewer 3) —***

Unless stated otherwise, all models below use the same five covariates as the primary ANCOVA (baseline SCC, self-esteem, trait self-reflection, generative AI experience, and age), scale scores computed from item-level responses, and 5,000 percentile bootstrap resamples (fixed seed) for indirect effects. Values are unstandardized coefficients with 95% confidence intervals.

**S14. Relative Indirect Effects for Each Control Condition (Revision)**

In the primary mediation models (Table 3), condition was coded GAISV = 1 versus the two image-control conditions combined = 0. To show the effect relative to each control condition, the three-category condition variable was entered with indicator coding (Hayes and Preacher, 2014), first with passive viewing and then with active non-self as the reference group.

*Table S14a. Relative indirect effects in the parallel mediation model.*

| **Time** | **Comparison** | **Indirect via CM** | **Indirect via flow** | **Contrast (CM − flow)** | **Direct effect** |
| --- | --- | --- | --- | --- | --- |
| T1 | GAISV vs. passive viewing | 0.117 [0.061, 0.188] | 0.015 [−0.015, 0.056] | 0.102 [0.029, 0.182] | 0.108 [−0.032, 0.247] |
| T1 | GAISV vs. active non-self | 0.106 [0.053, 0.174] | 0.003 [−0.009, 0.023] | 0.103 [0.048, 0.172] | 0.082 [−0.047, 0.213] |
| T1 | Active non-self vs. passive viewing | 0.011 [−0.039, 0.061] | 0.012 [−0.013, 0.044] | −0.001 [−0.058, 0.059] | 0.027 [−0.107, 0.162] |
| T2 | GAISV vs. passive viewing | 0.050 [0.003, 0.108] | 0.013 [−0.023, 0.058] | 0.037 [−0.030, 0.105] | 0.163 [0.007, 0.323] |
| T2 | GAISV vs. active non-self | 0.043 [0.002, 0.091] | 0.004 [−0.011, 0.025] | 0.039 [−0.006, 0.090] | 0.186 [0.034, 0.338] |
| T2 | Active non-self vs. passive viewing | 0.008 [−0.013, 0.038] | 0.009 [−0.016, 0.046] | −0.002 [−0.040, 0.036] | −0.023 [−0.164, 0.126] |

*Table S14b. Relative moderated mediation (moderator: image self-identification, standardized).*

| **Time** | **Comparison** | **Condition × ISI → CM** | **Index** | **Indirect, −1 SD** | **Indirect, mean** | **Indirect, +1 SD** |
| --- | --- | --- | --- | --- | --- | --- |
| T1 | GAISV vs. passive viewing | 0.418 [0.108, 0.732] | 0.085 [0.020, 0.168] | −0.095 [−0.222, 0.005] | −0.010 [−0.083, 0.060] | 0.075 [−0.007, 0.174] |
| T1 | GAISV vs. active non-self | 0.258 [−0.024, 0.556] | 0.052 [−0.005, 0.124] | −0.091 [−0.216, 0.010] | −0.039 [−0.112, 0.024] | 0.014 [−0.054, 0.084] |
| T2 | GAISV vs. passive viewing | 0.496 [0.158, 0.827] | 0.042 [−0.001, 0.114] | −0.039 [−0.122, 0.006] | 0.003 [−0.032, 0.041] | 0.046 [−0.002, 0.127] |
| T2 | GAISV vs. active non-self | 0.234 [−0.073, 0.534] | 0.020 [−0.005, 0.069] | −0.037 [−0.118, 0.006] | −0.017 [−0.062, 0.011] | 0.003 [−0.032, 0.046] |

The indirect effect through cognitive mirroring was reliable relative to each control condition at T1 and T2, and its advantage over the flow path was reliable relative to each control at T1. The two control conditions did not differ. The T1 index of moderated mediation was reliable relative to passive viewing but its confidence interval included zero relative to active non-self; neither T2 index excluded zero.

**S15. Mediation With Self-Description Only as the Baseline (Revision)**

GAISV (n = 80) was compared with the self-description-only condition (n = 82; T2: n = 76 and 70) in the same parallel mediation model. Participants in the self-description-only condition completed cognitive mirroring, flow, and post-task SCC but no image-specific measures, so the moderated mediation model, which requires image self-identification, could not be estimated with this condition as the baseline. Because the CM-HAIS was developed for responses to an AI image, the cognitive-mirroring comparison with this condition should be interpreted with caution.

*Table S15a. Parallel mediation: GAISV vs. self-description only.*

| **Path** | **T1** | **T2** |
| --- | --- | --- |
| GAISV → CM | 0.254 [0.021, 0.475] | 0.252 [0.002, 0.486] |
| GAISV → flow | 0.053 [−0.210, 0.301] | 0.054 [−0.217, 0.329] |
| CM → SCC | 0.269 [0.182, 0.357] | 0.100 [−0.016, 0.204] |
| Flow → SCC | 0.059 [−0.017, 0.136] | 0.017 [−0.081, 0.114] |
| Indirect via CM | 0.068 [0.005, 0.137] | 0.025 [−0.005, 0.069] |
| Indirect via flow | 0.003 [−0.016, 0.026] | 0.001 [−0.012, 0.021] |
| Contrast (CM − flow) | 0.065 [0.003, 0.136] | 0.024 [−0.012, 0.068] |
| Direct effect | 0.074 [−0.057, 0.207] | 0.138 [−0.024, 0.295] |

*Table S15b. Four-group model with self-description only as the reference group.*

| **Condition vs. self-description only** | **T1: condition → CM** | **T1: indirect via CM** | **T1: indirect via flow** | **T2: indirect via CM** |
| --- | --- | --- | --- | --- |
| GAISV | 0.253 [0.029, 0.473] | 0.062 [0.007, 0.124] | 0.003 [−0.010, 0.019] | 0.020 [−0.001, 0.054] |
| Active non-self | −0.202 [−0.408, 0.009] | −0.050 [−0.102, 0.002] | −0.000 [−0.015, 0.013] | −0.018 [−0.048, 0.003] |
| Passive viewing | −0.267 [−0.487, −0.043] | −0.065 [−0.124, −0.010] | −0.015 [−0.045, 0.006] | −0.025 [−0.063, −0.000] |

Relative to self-description only, GAISV showed a smaller but reliable T1 indirect effect through cognitive mirroring and no reliable T2 indirect effect. Cognitive mirroring after self-description only was higher than after passive viewing and directionally higher than after active non-self, indicating that writing about the self engaged some reflective processing on its own and that the AI-generated self-image added a further, more modest increment.
